# Transcriptional Profiling of Non-Small Cell Lung Cancer Cells with Activating *EGFR* Somatic Mutations

**DOI:** 10.1371/journal.pone.0001226

**Published:** 2007-11-21

**Authors:** Kuicheon Choi, Chad J. Creighton, David Stivers, Nobukazu Fujimoto, Jonathan M. Kurie

**Affiliations:** 1 Department of Thoracic/Head and Neck Medical Oncology, M. D. Anderson Cancer Center, The University of Texas, Houston, Texas, United States of America; 2 Dan L. Duncan Cancer Center, Baylor College of Medicine, Houston, Texas, United States of America; 3 Department of Bioinformatics and Computational Biology, M. D. Anderson Cancer Center, The University of Texas, Houston, Texas, United States of America; Max Planck Institute for Evolutionary Anthropology, Germany

## Abstract

**Background:**

Activating somatic mutations in *epidermal growth factor receptor* (*EGFR*) confer unique biologic features to non-small cell lung cancer (NSCLC) cells, but the transcriptional mediators of EGFR in this subgroup of NSCLC have not been fully elucidated.

**Methodology/Principal Findings:**

Here we used genetic and pharmacologic approaches to elucidate the transcriptomes of NSCLC cell lines. We transcriptionally profiled a panel of *EGFR*-mutant and -wild-type NSCLC cell lines cultured in the presence or absence of an EGFR tyrosine kinase inhibitor. Hierarchical analysis revealed that the cell lines segregated on the basis of *EGFR* mutational status (mutant versus wild-type), and expression signatures were identified by supervised analysis that distinguished the cell lines based on mutational status (wild-type versus mutant) and type of mutation (L858R versus Δ746-750). Using an *EGFR* mutation-specific expression signature as a probe, we mined the gene expression profiles of two independent cohorts of NSCLC patients and found the signature in a subset. EGFR tyrosine kinase inhibitor treatment regulated the expression of multiple genes, and pharmacologic inhibition of the protein products of two of them (*PTGS2* and *EphA2*) inhibited anchorage-independent growth in *EGFR*-mutant NSCLC cells.

**Conclusions/Significance:**

We have elucidated genes not previously associated with *EGFR*-mutant NSCLC, two of which enhanced the clonogenicity of these cells, distinguishing these mediators from others previously shown to maintain cell survival. These findings have potential clinical relevance given the availability of pharmacologic tools to inhibit the protein products of these genes.

## Introduction

Treatment with epidermal growth factor receptor (EGFR) tyrosine kinase inhibitors (TKIs) leads to rapid and sustained tumor shrinkage in a subset of patients with non–small cell lung cancer (NSCLC) [1]–[3]. The tumor cells in these patients have somatic mutations in the *EGFR* kinase domain that constitutively activate EGFR [1]–[3]. The activating mutations identified thus far cluster in the region that encodes the kinase domain (exons 18–21) and are most frequently either Δ746–750 deletion or L858R point mutations [1]–[3]. Mouse models constructed to investigate the oncogenicity of mutant *EGFR* develop invasive lung adenocarcinomas that regress after treatment with EGFR TKIs [4;5]. Immortalized human bronchial epithelial cells acquire malignant properties after transfection with mutant *EGFR* [6]. Treatment with EGFR TKIs induces apoptosis of these *EGFR*-transfected cells and NSCLC cells with somatic mutations in *EGFR* [7;8]. Thus, evidence from human, murine, and cellular models indicates that mutant *EGFR* is oncogenic and confers dependence on EGFR for NSCLC cell survival.

The potency of mutant *EGFR* as an oncogene is supported by evidence that its biochemical properties differ sharply from those of wild-type EGFR. The EGFR kinase domain is constitutively activated by the somatic mutations, and it displays enhanced binding and sensitivity to EGFR TKIs [9]–[11]. *EGFR*-mutant NSCLC cells typically express high levels of EGFR and its dimeric partners ErbB2 and ErbB3 and multiple ErbB ligands, all of which potentiate EGFR-dependent signaling [8]. Multiple signaling pathways are constitutively activated in these cells, some of which have been shown to promote cell survival. For example, EGFR forms a heterodimeric complex with ErbB3, which binds to and directly activates phosphatidylinositol 3-kinase and maintains cell survival through AKT-dependent mechanisms [12]. Other pro-survival signals are mediated through Src family kinases, which are constitutively activated in *EGFR*-mutant NSCLC cells [13;14].

In contrast to the advances made in elucidating mediators of cell survival, less progress has been made in understanding the mechanisms by which mutant *EGFR* confers other neoplastic properties, such as the ability to migrate, invade, proliferate in an anchorage-independent manner, and promote angiogenesis. Here we sought to identify those mediators by interrogating the transcriptomes of a panel of NSCLC cell lines that have been characterized for the presence of *EGFR* mutations. We found a transcriptional profile of *EGFR*-mutant NSCLC cells that included genes not previously been known to be EGFR-dependent. Although the range of cellular functions attributed to these genes is broad, many of them are linked through known or predicted protein interaction networks. In conclusion, the transcriptional profile identified in *EGFR*-mutant NSCLC cells has informed us about biologic processes and potential therapeutic targets that could be effective in patients with this disease.

## Results

### Transcriptional Analysis of NSCLC Cell Lines

We used a panel of eight NSCLC cell lines (Table 1) that had been characterized for the presence or absence of somatic *EGFR* mutations (five cell lines with such mutations and three without) and *Ras* mutations (two cell lines with such mutations and six without). Of the five *EGFR*-mutant cell lines, three had exon 19 deletion mutations (Δ746–750) (HCC827, HCC2279, H4006), one had an exon 21 point mutation (L858R) (H3255), and one had L858R in combination with the T790M gatekeeper mutation that confers resistance to EGFR TKIs (H1975). Of the three *EGFR*-wild-type cell lines, one had a somatic mutation in *N-ras* (H1299), and one had a *K-ras* mutation (H460). RNA was extracted and prepared from cells after they had been cultured for 2 h at 70% confluence in the presence or absence of the EGFR TKI gefitinib (1.0 µM). This duration of TKI treatment was chosen to identify the earliest transcriptional events induced by EGFR inhibition and to minimize the detection of gene expression changes due to apoptosis. The RNA was subjected to Affymetrix gene expression profiling, and the profiles were examined for differences in gene expression at baseline and after TKI treatment.

**Table 1:** Characteristics of NSCLC Cell Lines

Cell Line	EGFR	Gefitinib IC_50_	Ras
H1299	WT	38.0 µM	N-RasQ61K
H1819	WT	4.7 µM	WT
H460	WT	8.0 µM	K-RasQ61H
H1975	L858R, T790M	1.9 µM	WT
HCC2279	Δ746–750	5.0 µM	WT
H3255	L858R	9.0 nM	WT
H4006	Δ746–750	30.0 nM	WT
HCC827	Δ746–750	16.0 nM	WT

Abbreviations: WT, wild-type; IC_50 _, 50% inhibitory concentration Gefitinib IC_50 _values have been reported (Fujimoto et al., 2005).

**Figure 1:**
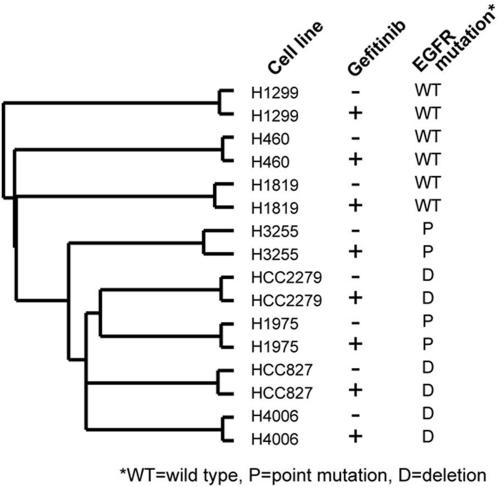
Hierarchical analysis of gene expression profiles in eight NSCLC cell lines. Dendrogram illustrating the relatedness of expression profiles from cell lines treated with (+) or without (−) gefitinib. The presence or absence of *EGFR* somatic mutations and the type of mutations, including L858R point mutation (P) or Δ746–750 deletion mutation (D), are indicated. WT, wild-type.

**Figure 2:**
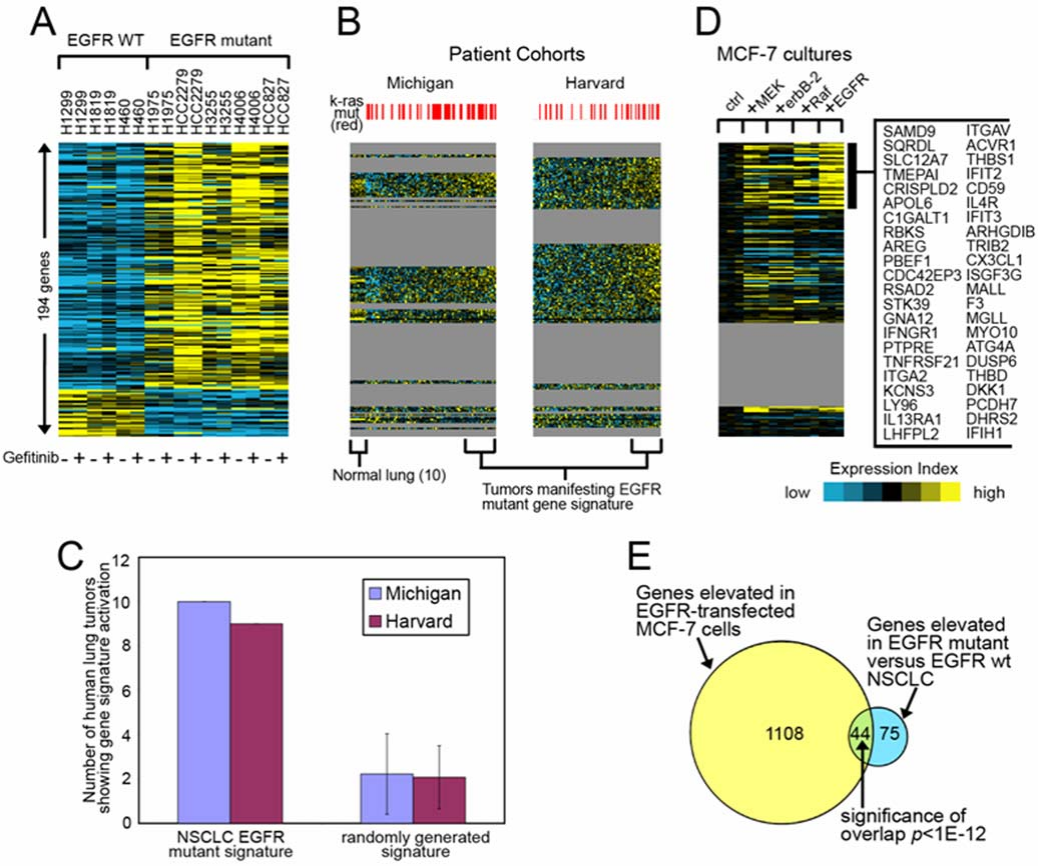
Identification of a mutant *EGFR* gene expression profile. *(A)* A 194-gene signature that distinguishes *EGFR*-wild-type (WT) from -mutant NSCLC cell lines (treated with [+] or without [−] gefitinib) is aligned with the expression profiles from *(B)* the Michigan cohort (86 patients) and the Harvard cohort (84 patients) and *(D)* MCF-7 cells transfected with the indicated genes. A list of the genes that overlapped in all three data sets is indicated on the far right. (*C*) Numbers of patients in the Michigan and Harvard cohorts manifesting the mutant *EGFR* expression patterns (*P*<0.05, Pearson's correlation), along with the number manifesting a randomly generated pattern (SD based on 100 simulations). (*E*) Venn diagram illustrating the overlap between signatures from the *EGFR*-mutant NSCLC cells and *EGFR*-transfected MCF-7 cells.

We first examined *EGFR* mutational status as a classifier of the transcriptional profiles. Hierarchical analysis revealed clustering of the cell lines based on mutational status; the *EGFR*-mutant cells lines clearly segregated from the -wild-type cell lines (Fig. 1). However, there was no clear separation between the two types of mutations (L858R versus Δ746–750) (Fig. 1). By supervised analysis using specific criteria (at least a 2.0-fold increase or a 50% decrease in *EGFR*-mutant cell lines relative to that of wild-type, *P*<0.05), 194 unique genes were identified that distinguished the *EGFR-*mutant cell lines from the –wild-type cell lines. These genes are listed in File S1 and illustrated in a clustered heat map in Fig. 2*A*. We examined 29 of the 194 genes by quantitative PCR and validated that 19 (68%) of them were differentially expressed between *EGFR*-mutant and –wild-type cell lines (File S2).

### Identification of the Mutant *EGFR* Signature in Cohorts of Patients with NSCLC

We next queried publicly available gene expression databases of tumor biopsy samples derived from two independent cohorts of patients with NSCLC from the United States (15, 16) to determine whether a subset of tumors expressed this genetic signature. Of the 194 genes, 102 (53%) were represented on the profiling platform used in the Harvard cohort, and 65 (34%) were represented in the Michigan cohort. Based on a heat map representation of their gene expression patterns, the human lung tumors were heterogeneous in their expression patterns; however, a subset of tumors (9 of 84 [11%] in the Harvard cohort and 10 of 86 [12%] in the Michigan cohort) exhibited an expression pattern similar to that of the *EGFR*-mutant NSCLC gene signature (Fig. 2*B*).

To determine whether the similarities observed by heat map analysis reached statistical significance, parameters were created that defined similarity as a positive Pearson's correlation of *P*<0.05 (two-sided) between the mutant *EGFR* signature pattern and the gene expression values of the tumor, taking into account both the over- and under-expressed genes in the signature. By this definition, tumors manifesting this signature would recapitulate the patterns of over-and under-expression observed in the *EGFR*-mutant cell lines. The incidence of tumors manifesting the signature was statistically significant (*P*<0.01 for each cohort) based on simulations using 100 randomly selected gene signatures generated from each of the cohorts (Fig. 2C).

Although the *EGFR* mutational status of tumors from these patient cohorts has, to our knowledge, not been reported, *K-ras* (codons 12, 13, or 61) is known to be mutated in 29% and 45% of the tumors from the Harvard and Michigan cohorts, respectively [15;16]. Somatic mutations in *EGFR* and *K-ras* are mutually exclusive in NSCLC [17], which has led investigators to hypothesize that these events are genetically redundant and that a change in both genes does not confer a further advantage when these events occur together in the same cell [17]. We postulated that, if these somatic events are genetically redundant, then having the *K-ras* mutation will confer the mutant *EGFR* transcriptional profile. Consistent with this idea, we noted that *K-ras* mutational status correlated, albeit weakly, with the mutant *EGFR* gene signature (Fig. 2*B, P* = 0.07 and *P* = 0.04 in the Harvard and Michigan cohorts, respectively, by Wilcoxon rank-sum tests of the cohort profiles ordered by average expression of genes that were increased in *EGFR*-mutant cell lines).

### Mutant *EGFR* Signature Enriched with EGFR-Dependent Genes

To investigate whether any of the genes in the 194-gene signature were regulated in an EGFR-dependent manner, we queried a publicly available database of MCF-7 breast cancer cells that had been stably transfected with constitutively active kinases (*Raf1*, *MEK1*, *ErbB2*) or with wild-type *EGFR*, which was activated by short-term EGF treatment [18]. We investigated the overlap between the gene signatures from *EGFR*-transfected MCF-7 cells and *EGFR*-mutant NSCLC cells. Of the 194 genes in the mutant *EGFR* signature, 139 (72%) were represented on the profiling platform for the MCF-7 cell transfectants (11079 genes in all were represented in both the MCF-7 and NSCLC datasets). Analysis of these 139 genes revealed that subsets of the genes that were increased in MCF-7 cells because of MEK, Raf1, or EGFR transfection overlapped with those in the mutant *EGFR* expression signature in NSCLC cells (Fig. 2*D*).

Of the 119 genes that were both represented in the MCF-7 dataset and increased in *EGFR*-mutant NSCLC cells, 44 (31%) were increased with *P*<0.05 in *EGFR*-transfected MCF-7 cells, which represented a highly significant overlap (*P*<1E–12, one-sided Fisher's exact test, Fig. 2*E*). By chance, 14 of the 119 genes would be expected to overlap, indicating that the amount of overlap we observed exceeded what would be expected if the *EGFR*-transfected MCF-7 cells and *EGFR*-mutant NSCLC cells nothing biologically in common with each other. When we used a more stringent cut point of *P*<0.01 instead of *P*<0.05 to define genes that were increased in *EGFR*-transfected MCF-7 cells (576 genes in all), 28 overlapped with the mutant EGFR NSCLC signature (chance expected of seven genes, *P<*1E–10, one-sided Fisher's exact test). We concluded that, based on its commonalities with the signature from *EGFR*-transfected MCF-7 cells, the signature from *EGFR*-mutant NSCLC cells was enriched for genes transcriptionally up-regulated by EGFR.

### Identification of *PTGS2* as a gene required for anchorage-independent growth

The genes that were differentially expressed in *EGFR*-mutant NSCLC cell lines fell into a broad range of functional classes (categorized in the Gene Ontology Molecular Functions, www.geneontology.org) (Files S3 and S4). The categories with highest representation were signal transduction, metabolism, immune response, ion transport, and cell cycle and proliferation. Genes in these categories that were highly expressed included those encoding ErbB ligands (*TGFA, AREG*, and *EREG*), cyclooxygenase-2 (*PTGS2*), a ligand for the CX3CR1 chemokine receptor (*CX3CL1)*, intracellular and transmembrane kinases (*TRIB2, MET, MYLK, STK39, ACVR1, TAOK3, and IFIH1*), protein phosphatases (*PTPN22, DUSP10, PPAP2B, PTPRR,* and *PTPRE*), a lipid phosphatase (*SGPP2*), adhesion molecules (*CEACAM6, ITGAV, PCDH7,* and *THBS1*), and calcium ion-binding proteins (*S100A14* and *S100A16*).

To examine whether these differentially expressed genes, which have disparate biologic functions, were part of one or more signal transduction networks, we analyzed their positions within known or predicted global protein interaction networks (interactomes) using the HiMAP software program (http://www.himap.org/index.jsp). Interactomes identified by this approach are organized into a series of modular structures characterized by centrally-located nodes (called hubs) that have multiple connections with other proteins [19]. Although this approach is purely exploratory and carries no statistical weight, findings in yeast show that centrality in a protein interactome predicts a protein's biological importance [20].

**Figure 3:**
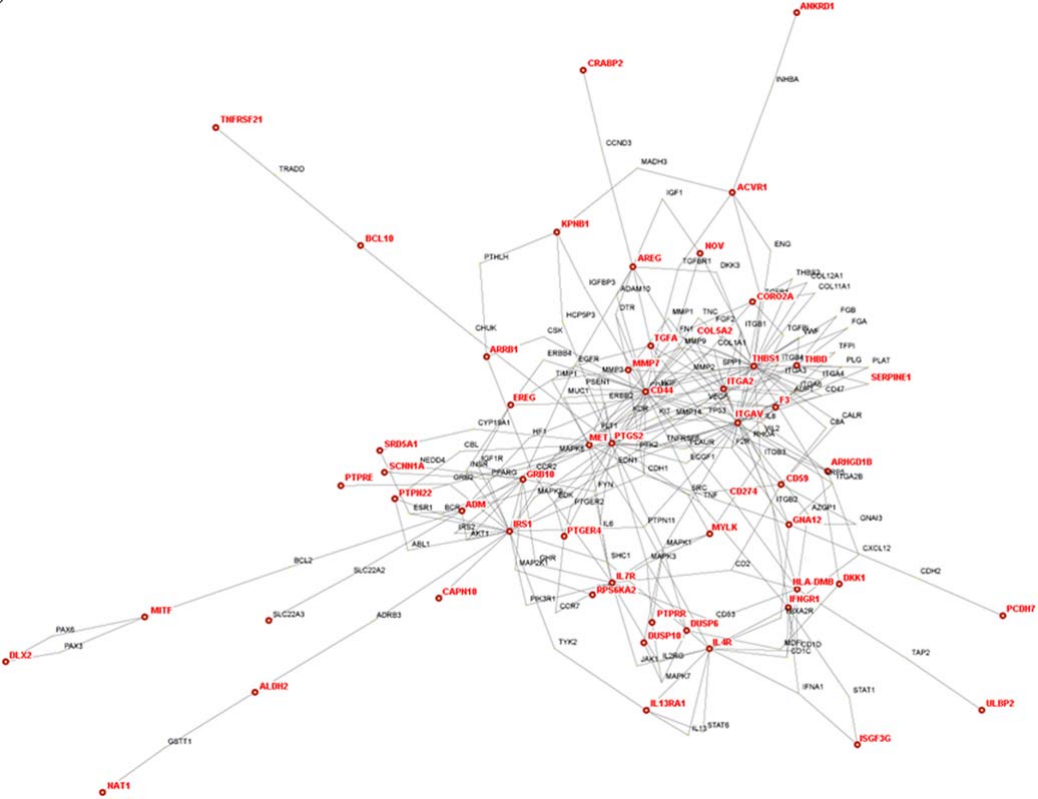
Interactome of genes in the mutant EGFR expression signature. Theoretical protein-protein physical and functional interaction map (interactome) was drawn using HiMAP software. Genes from the signature are indicated in red.

Of the 194 differentially expressed genes, 118 had been annotated in Gene Ontology, of which 102 were included in the HiMAP software program (19). Of those 102 genes, 52 mapped within a single network (Fig. 3). The hubs in the network with the highest numbers of links (≥ 10) included *CD44, MET, IRS1, GRB10, ITGA2, PTGS2, and THBS1,* all of which were highly expressed in *EGFR*-mutant NSCLC cells, indicating that some of the differentially expressed genes were at central positions of this signaling network.

**Figure 4:**
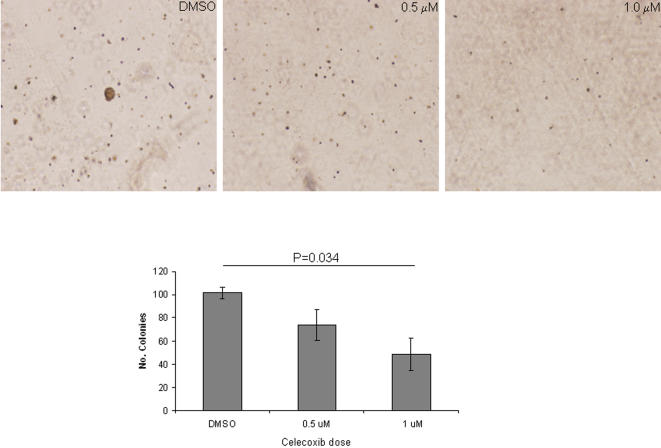
Cyclooxygenase-2 inhibition decreases NSCLC anchorage-independent growth. Representative images of colonies of NSCLC cell lines (upper panels) were quantified (lower panels) after growing them in soft agar in the presence or absence of celecoxib. Results are the means of at least three independent experiments.

In light of the centrality of the *PTGS2* gene in the interactome network and the known importance of its gene product cyclooxygenase-2 (COX-2) in the survival and metastasis of cancer cells and its potential clinical impact given the availability of pharmacologic tools to inhibit its enzymatic function [21], we sought to investigate its role in *EGFR*-mutant NSCLC cells. We examined whether the cyclooxygenase-2 inhibitor celecoxib abrogated the ability of these cells to proliferate in monolayer cultures and to form colonies in soft agar. Using celecoxib at low doses (0.5 µM and 1.0 µM) to minimize nonspecific effects, colony formation in soft agar decreased in a dose-dependent manner (Fig. 4), whereas proliferation in monolayers did not change (data not shown).

### Cell Lines with *EGFR* Deletions and Point Mutations Have Distinct Expression Profiles

**Figure 5:**
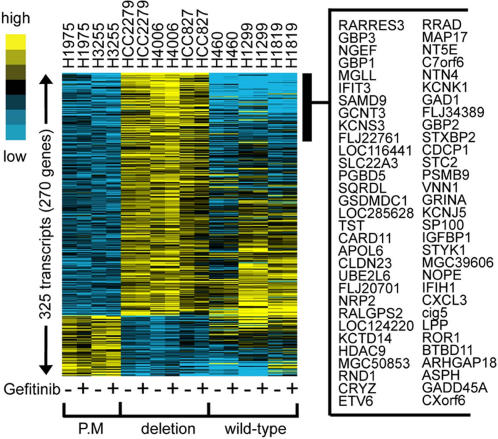
Expression signature that distinguishes two types of *EGFR* mutations. A 194-gene signature present in *EGFR*-mutant but not EGFR–wild-type cell lines distinguishes L858R (P.M. [point mutation]) from Δ746–750 (deletion). Cells were treated with (+) or without (−) gefitinib. A partial list of the genes that are differentially expressed in the two groups is indicated on the right.

Among patients with *EGFR*-mutant NSCLC, differences in survival duration and responsiveness to EGFR TKI treatment have been observed depending on the type of *EGFR* somatic mutation (exon 21 point mutations versus exon 19 deletions) [22;23], suggesting that these two types of somatic mutations confer distinct biologic properties to NSCLC cells. Supporting this possibility is evidence that these two types of somatic mutations confer distinct biochemical properties to EGFR [9]–[11]. Although hierarchical clustering did not segregate the two types of mutations into two distinct subgroups (Fig. 1), we hypothesized that supervised analysis would reveal transcriptional differences between the two types of mutations. Indeed, clear transcriptional differences were observed. Using specific criteria (*P*<0.01, at least two-fold change), we identified a 270-gene signature in *EGFR*-mutant cell lines (which was not present in *EGFR*-wild-type cell lines) that distinguished the two types of mutations. These genes are listed in File S5 and illustrated in a clustered heat map in Fig. 5. We examined 7 of the 270 genes by quantitative PCR and validated that 4 (57%) were differentially expressed between cell lines with L858R mutations versus those with Δ746–750 mutations (File S6). The 270-gene signature was analyzed for enrichment in specific gene functions as defined by the Gene Ontology Signature Database. L858R-mutant cells were enriched for genes in the cyclic AMP-dependent protein kinase- , protein phosphatase-2ε-, Ras-family member RAB3D-, and phospholipase-Cβ1-dependent pathways, whereas the Δ746–750 mutants were enriched for genes in the CXC chemokine ligands (2 and 3)-, integrin α6-, guanylate binding proteins (1, 2, and 3)-, and interleukin-7 and 10-dependent pathways (File S7), demonstrating that the gene sets activated by the two mutation types are functionally distinct.

### Genes Regulated by EGFR TKI Treatment

To identify gene expression changes that preceded, and possibly contributed to, the biologic effects of EGFR TKI treatment on *EGFR*-mutant NSCLC cells, the cells were subjected to short-term treatment with gefitinib. As a negative control in this experiment, we used the TKI-resistant H1975 cells, which have a T790M mutation that blocks binding to the EGFR TKI [17]. Using specific criteria (*P*<0.05), we found that 54 genes were regulated by TKI exclusively in the TKI-sensitive cell lines (HCC827, H3255, and H4006) (File S8), none of which have, to our knowledge, been reported to be EGFR-dependent genes. Among these genes, we examined 14 by quantitative PCR and validated that 10 (71%) were differentially regulated in TKI-sensitive and resistant cells (File S9). Genes that increased in response to TKI included, among others, cell cycle regulators (*CCNG2, CDKN1B, ID2, and KNTC2*) and a ligand for EphA2 (EFNA1). Genes that decreased include the *EphA2* receptor tyrosine kinase, cytokines (*LIF, CCL20,* and *IL17B*), transcription factors (*FOXD1* and *POU1F1*), and protein phosphatases (*DUSP4* and *DUSP6*).

### Reciprocal Regulation of EphA2 and EFNA1 is EGFR-Dependent

**Figure 6:**
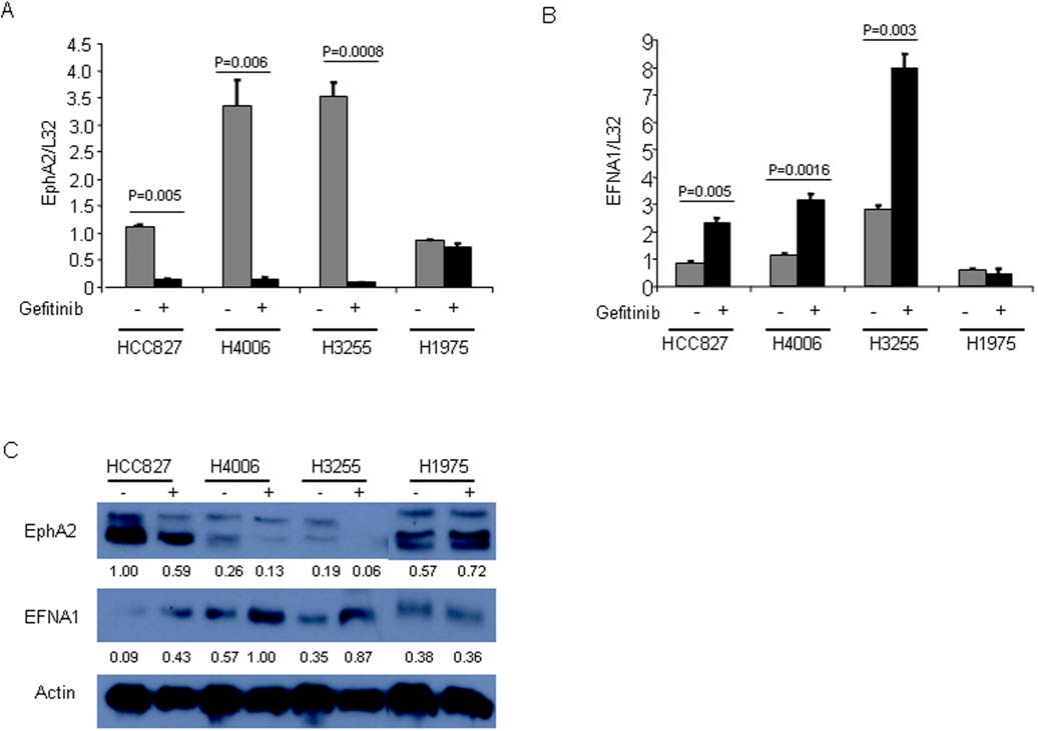
Reciprocal regulation of EphA2 and EFNA1 in NSCLC cell lines. Quantitative PCR analysis *(A, B)* and western blotting *(C)* of EphA2 *(A, C)* and EFNA1 *(B, C)* in cell lines treated for 6 h with (+) or without (−) gefitinib. Quantitative PCR results represent the means of at least three independent experiments and were normalized based on expression of the housekeeping gene L32. The numbers under the bands are the results of densitometric analysis after normalizing for loading differences based on actin.

**Figure 7:**
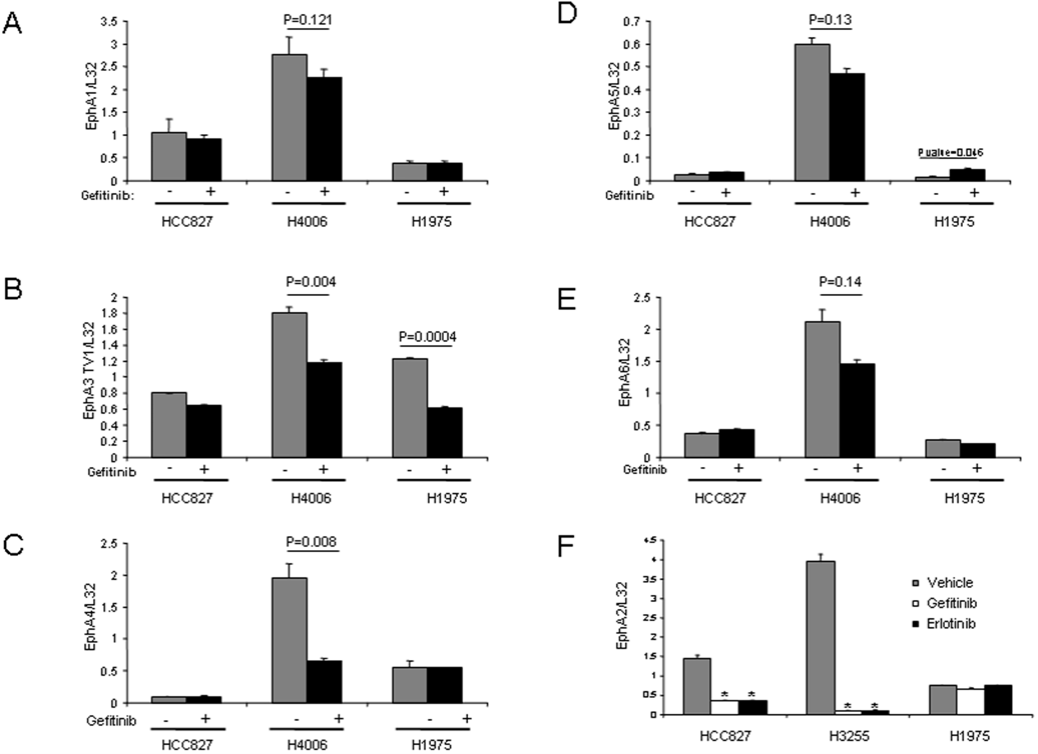
EGFR TKI-induced changes in expression of EphA family members. Cells were treated for 6 h with vehicle, 1 µM gefitinib (A–E), or 1 µM erlotinib (F). RNA was extracted and subjected to quantitative PCR. Results represent the means of at least three independent experiments and were normalized based on L32 expression.

In light of the importance of the EphA axis in tumorigenesis [24;25], we further investigated the effects of TKI treatment on the expression of EphA2, other EphA family members, and their ligand EFNA1. Quantitative PCR and western analysis confirmed that gefitinib reciprocally regulated the expression of EphA2 and its ligand EFNA1 in TKI-sensitive cell lines (HCC827, H4006, and H3255) but not in the TKI-resistant H1975 cells (Fig. 6*A–C*). EphA1, EphA5, and EphA6 did not change with gefitinib treatment (Fig. 7*A, E, and F*); EphA4 decreased in only a subset of TKI-sensitive cells (H4006 but not HCC827) (Fig. 7*C*); and EphA3 was inhibited in an EGFR-independent manner (indicated by the TKI-induced suppression in H1975 cells) (Fig. 7*B*). To more fully evaluate whether the suppression of EphA2 expression was EGFR-mediated, we examined the effect of another EGFR TKI, erlotinib, and found that it too inhibited EphA2 expression in TKI-sensitive cells, to an extent similar to that of gefitinib (Fig. 7*F*).

### EphA2 Activation is Required for Anchorage-Independent Growth

**Figure 8:**
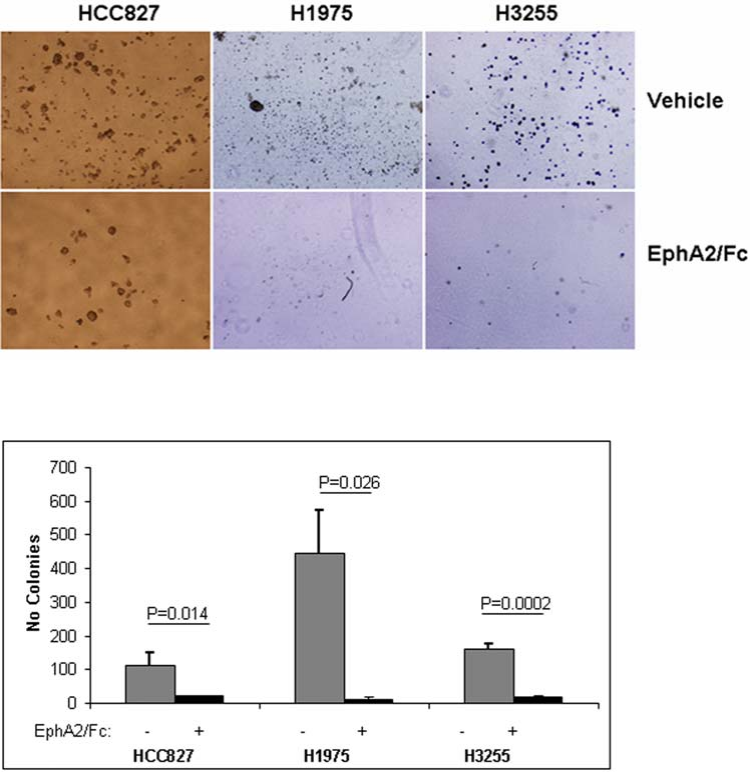
EphA2-Fc inhibits NSCLC anchorage-independent growth. Representative images of colonies of NSCLC cell lines (top) with quantification of colonies (bottom) after growth in soft agar in the presence or absence of EphA2-Fc. Results are the means of at least three independent experiments.

We postulated that EphA2 signaling maintains neoplastic features of NSCLC cells and tested this hypothesis by treating HCC827 cells with EphA2-Fc, a recombinant peptide containing the EphA2 extracellular domain fused to the F_c_ fragment of IgG, which prevents the interaction of ephrin A ligands with endogenous EphA, effectively blocking EphA activation [26]. Relative to that of controls, EphA2-Fc-treated cells exhibited decreased colony formation in soft agar (Fig. 8) whereas their proliferation in monolayer cultures did not change (data not shown), indicating that EphA was required for the anchorage-independent proliferation of these cells.

## Discussion

Here we report that NSCLC cells with somatic *EGFR* mutations have a unique transcriptional profile and that cell lines with the two most common types of *EGFR* mutations have clear transcriptional differences. By mining gene expression databases using a mutant *EGFR*-specific signature as a probe, we found that many of the genes in this expression signature were EGFR-dependent, converged into common networks on the basis of known or predicted protein interactomes, and were expressed in tumors from a subset of patients with NSCLC. Two genes were elucidated, *EphA2* and *PTGS2*, that promoted the clonogenicity of *EGFR*-mutant NSCLC cells, which are of particular interest from a clinical standpoint because they can be inhibited pharmacologically.

Genes within the mutant *EGFR* gene expression signature encode proteins with a diverse set of cellular functions. The influence of EGFR on this signature was demonstrated by its overlap with that of EGFR-transfected MCF-7 cells and the presence of known EGFR transcriptional targets, including *PTGS2,* the ErbB ligands *EREG* and *AREG,* and *Met*, a receptor tyrosine kinase that was recently reported to be activated in *EGFR*-mutant NSCLC cells and to promote TKI resistance in these cells [8;27–29]. Here we showed that the gene product of *PTGS2*, cyclooxygenase-2, has an important role, promoting anchorage-independent growth. This signature contained many genes not previously known to be highly expressed in *EGFR*-mutant NSCLC, including *LY96* and *CX3CL1*, which have known immunomodulatory functions. *LY96* encodes MD2, an accessory molecule required for the activation of toll-like receptor-4, which promotes cell survival and induces the secretion of immunosuppressive molecules that promote tumor evasion from immune surveillance [30]. *CX3CL1* encodes a secreted protein called fractalkine that recruits CX3CR1-expressing natural killer and T lymphocytes to the tumor microenvironment, thereby promoting natural killer-dependent antitumor responses *in vivo* [31]. On the other hand, fractalkine has also demonstrated pro-metastatic properties based on evidence that it promotes tumor cell migration and enhances adhesion of tumor cells to endothelial cells [32;33].

To identify genes regulated in an EGFR-dependent manner, we treated *EGFR*-mutant NSCLC cells with gefitinib. Two of the genes identified by this approach, *EphA2* and its ligand *EFNA1*, were regulated in a reciprocal fashion. Potentially mediating this effect of gefitinib, mitogen-activated protein kinase, a downstream effector of EGFR, inhibits *EFNA1* expression, thereby relieving the EFNA1-induced suppression of *EphA2* expression [25]. Moreover, we found that EphA2/EFNA1 interactions were required for the anchorage-independent growth of HCC827 cells, which corroborates findings from a previous study demonstrating that v-ErbB-dependent cellular transformation is attenuated by EphA2 ligand-binding [25]. Other genes we found to be regulated by gefitinib in TKI-sensitive cells include *CCNG2, CDKN1B, ID2, and KNTC2*, which are components of cell cycle regulatory pathways. Given that their expression changed before any biochemical evidence of proliferative arrest or apoptosis, these genes might be part of an anti-proliferative signaling program activated by gefitinib. Lastly, two of the genes that decreased in abundance with gefitinib treatment (*CEACAM-6* and *DUSP6*) were also highly expressed in *EGFR*-mutant cells, suggesting that these genes are potentially important EGFR transcriptional targets in these cells.

In summary, we have identified a transcriptome in NSCLC cells that elucidates mutant *EGFR*-induced gene expression changes and provides a transcriptional basis for the biologic differences observed in NSCLC with the two most commonly occurring types of *EGFR* mutations. Further analysis of these genes may inform us about biologic processes that can be used to identify intracellular targets of potential therapeutic benefit for patients with this disease.

## Materials and Methods

### Reagents

Gefitinib (Astra Zeneca Pharmaceuticals, Wilmington, DE) and erlotinib (OSI Pharmaceuticals, Melville, NY) were gifts. We purchased a recombinant murine EphA2-Fc chimera (R&D Systems, Minneapolis, MN), polyclonal antibodies derived in rabbits against EphA2 and EFNA1 (Santa Cruz Biotechnologies, Santa Cruz, CA), a horseradish peroxidase–linked anti-mouse and ant-rabbit secondary antibodies (Cell Signaling Biotechnology, Beverly, MA), and an antibody against β-actin (Sigma-Aldrich, St. Louis, MO).

### Cell Lines

The NSCLC cell lines used in this study were purchased from the American Type Culture Collection (Manassas, VA) and were grown in 5% CO_2_ at 37°C in RPMI 1640 medium with high glucose (4.5 g/L; GIBCO-BRL, MD), supplemented with 10% fetal bovine serum (Hyclone, Logan, Utah).

### Gene Expression Profiling

RNAs were isolated by using the RNeasy Mini Kit (Qiagen, Valencia, CA) and hybridized to Affimetrix U133+2.0 gene expression chips at the M.D. Anderson Cancer Center Microarray Core Facility (supported in part by grant CA #16672). dChip (http://www.dchip.org) (2005 version) was used to extract expression values for each probe set. Control probe sets and probe sets with suffixes “_s_at” and “_x_at” on their ID were excluded (because these probe sets may target more than one unique sequence), leaving 39,114 of 54,675 original probe sets for further analysis.

### Determination of differentially expressed genes

Two-sample *t* tests (using log-transformed data) were used to determine significant differences in gene expression between *EGFR*-mutant and -wild-type NSCLC cell lines and between EGFR-transfected and parental MCF-7 cells (*P* values were two-sided). For the analysis of gefitinib treatment effects, a paired difference was calculated as the log_2_(gefitinib-treated/vehicle-treated).

### GO enrichment analysis

Functional gene groups as defined by Gene Ontology (GO) annotation (http://www.geneontology.org) were evaluated for enrichment within our own experimentally-derived gene sets. Essentially as described in [34], one-sided Fisher's exact tests were performed to assess whether or not genes in each GO functional group were over-represented in our gene set (the 16,172 genes represented by the 39,114 probe sets on the array were used as the reference population). Gene Ontology annotations were obtained from the NCBI's annotation file gene2go (ftp://ftp.ncbi.nlm.nih.gov/gene/DATA). In addition to one-sided Fisher's exact *P*-values, *Q*-values were computed to account for multiple term testing, using the method by Storey *et al*. [35].

### HiMAP interactome analysis

Gene lists were imported into the HiMAP program (http://www.himap.org/index.jsp) for protein-protein interaction network analysis. HiMAP (19) includes both experimentally-validated protein-protein interactions (as cataloged in the Human Protein Reference Database, or HPRD [www.hprd.org]), and predicted protein-protein interactions based on a probabilistic model integrating multiple factors, including interactome data from the Database of Interacting Proteins [36], protein domain data, genome-wide expression data, and functional annotation data from GO.

### Analysis of the EGFR mutation gene signature in additional profile datasets

Expression values were visualized as color maps using the Cluster and Java TreeView programs [37; 38]. Expression values in the human lung adenocarcinoma datasets were transformed to standard deviations from the tumor mean. The set of profiles analyzed in the Harvard dataset were the same set as was analyzed in Beer et al. (15). For correlating the human NSCLC tumor profiles with the mutant *EGFR* gene signature (Figure 2C), +1 and -1 were used to represent each of the genes up and down, respectively, in the signature. The Pearson's correlation coefficient was computed based on the comparison of the mutant *EGFR* signature pattern to that of each human NSCLC tumor profile. In 100 separate simulation tests, a randomly generated gene signature (with the same number of up and down genes as there was in the mutant *EGFR* signature) was generated, and the Pearson's correlation was computed based on the comparison of this random signature to each human NSCLC tumor. In no single test did the number of tumors with significant positive correlations (p<0.05, two-sided) to the random pattern exceed the number that correlated positively with the mutant *EGFR* signature.

The Entrez Gene identifier was used for mapping genes from the NSCLC cell line dataset to those from the human NSCLC tumor and MCF-7 profile datasets. Where a gene was represented several times on a given platform, an appropriate rule was used to select the “best” gene probe in a manner not biased towards detecting patterns of concordance between datasets (for human lung tumor datasets, the probe with the greatest variation; for MCF-7 datasets, the probe with the greatest difference in either direction by *t* test between EGFR and control). As an alternative approach to exclude potential bias, overlap between the NSCLC and MCF-7 data sets was examined using probes randomly selected from the NSCLC expression arrays, which did not qualitatively affect the results reported.

### Quantitative PCR

The level of mRNA for each gene was measured with SYBR-Green–based real-time PCR. The primers used for real-time PCR were designed by using Primer Express (Applied Biosystems, Foster City, CA). The primer sequences used are listed in File S10. Each cDNA sample (7 µl) was amplified by using SYBR Green PCR Master Mix according to the manufacturer's instructions. The PCR products and their dissociation curves were detected with the 7500 Fast Real-Time PCR System (Applied Biosystems). The level of the housekeeping gene Ribosomal gene *Rpl32* (L32) in each sample was used as an internal control.

### Western Blotting

Cells were lysed with M-PER Mammalian Protein Extraction Reagent (Pierce, Rockford, IL). Lysates were cleared by centrifugation and protein concentrations were quantified with 1X Quick Start Bradford Dye Reagent (Bio-Rad Laboratories, Hercules, CA) so that equal amounts of protein (40 µg) could be resolved on 10% SDS-polyacrylamide gels. After transfer to membranes, samples were processed and visualized with ECL Western Blotting Reagents (Amersham Biosciences, Piscataway, NJ). All of the Western blotting data shown in this study are representative of at least three independent experiments.

### Anchorage-Independent Growth Assay

A bottom layer of agar was prepared in 60-mm wells by using 3 ml of 1% low melting temperature agarose in normal growth medium. Next, 3 ml of 0.5% low melting temperature agarose in normal growth medium containing 1×10^5^ cells was added on top of the solidified bottom layer. Every 3 days, 5 µg of EphA2-Fc dissolved in normal growth medium was added to each plate. Colonies showing anchorage-independent growth were counted 10–30 days later.

### Cell Viability Assay

Cell viability was measured with CCK-8 (Dojindo Molecular Technologies, Gaithersburg, MD) according to the manufacturer's protocol, in 96-well plates (seeding density, 5,000 cells per well) at 3 days after treatment with 5 µg/ml of EphA2-Fc or 0.5 µM and 1.0 µM of celecoxib.

## Supporting Information

File S1 Comparison of Gene Expression in EGFR-mutant and -wild-type NSCLC Cells. Genes listed were increased or decreased in EGFR-mutant NSCLC cell lines (n = 4) relative to that of EGFR wild-type cell lines (n = 3).
(0.06 MB XLS)

File S2 Quantitative PCR Analysis of Selected Genes that were Differentially Expressed Based on Expression Profiling of EGFR-mutant and -wild-type NSCLC Cell Lines. Results normalized based on L32 ribosomal RNA expression.
(0.07 MB DOC)

File S3 Mutant EGFR expression profile includes genes with diverse functions. Differentially expressed genes were grouped based on their Gene Ontology functions and represented in a pie chart to illustrate their relative abundance.
(0.07 MB TIF)

File S4 Mutant EGFR expression profile includes genes with diverse functions. Differentially expressed genes are listed according to their Gene Ontology functions.
(0.43 MB XLS)

File S5 Comparison of gene expression in EGFR L858R versus del746-750 NSCLC Cells. Genes listed were increased or decreased in EGFR L858R NSCLC cell lines (n = 2) relative to that of EGFR del746-750 cell lines (n = 3).
(0.08 MB XLS)

File S6 Quantitative PCR analysis of selected genes that were differentially expressed in EGFR L858R and δ746-750 NSCLC Cell Lines. Results normalized based on L32 ribosomal RNA expression.
(0.08 MB DOC)

File S7 Gene Ontology terms enriched in NSCLC cell lines with EGFR L858R and del746-750.
(0.50 MB XLS)

File S8 Comparison of gefitinib-induced gene expression changes in TKI-sensitive and -resistant EGFR-mutant NSCLC cell lines. The fold difference was calculated by log2[Gefitinib-induced change in expression in sensitive cells (HCC827, H3255, H4006)/resistant cells (H1975)].
(0.03 MB XLS)

File S9 Quantitative PCR analysis of selected genes that were regulated by gefitinib treatment in TKI-sensitive NSCLC cells
(0.09 MB DOC)

File S10 Primers used for quantitative PCR
(0.03 MB XLS)
