# Supplementary material for: Transcriptional Profiling of Non-Small Cell Lung Cancer Cells with Activating EGFR Somatic Mutations
Source: PLoS One. 2007 Nov 21;2(11):e1226. doi: 10.1371/journal.pone.0001226 (PMC2080626; doi:10.1371/journal.pone.0001226)
Supplement: File S2 — Quantitative PCR Analysis of Selected Genes that were Differentially Expressed Based on Expression Profiling of EGFR-mutant and -wild-type NSCLC Cell Lines. Results normalized based on L32 ribosomal RNA expression. (0.07 MB DOC) [file pone.0001226.s002.doc]

File S2. Quantitative PCR Analysis of Selected Genes that were Differentially Expressed Based on Expression Profiling of *EGFR*-mutant and –wild-type NSCLC Cell Lines

***(EGFR*-mutant)**

**(*EGFR*-wild-type)**

**H4006**

**HCC8277**

**H460**

**H1299**

8.000

0.526

0.070

0.070

THBS1

9.640

0.277

0.001

0.001

CEACAM6

3.791

2.825

0.615

0.425

IFI30

5.929

3.906

0.070

0.070

CX3CL1

6.817

3.054

0.070

0.070

F3

1.904

6.516

0.070

0.070

TNFRSF21

2.131

0.604

0.060

0.000

GRB10

0.649

3.289

0.032

0.008

LY96

1.581

2.610

0.439

0.305

MET

3.140

2.780

0.053

0.650

IL4R

14.297

1.560

0.029

0.015

IL7R

5.345

1.670

0.003

0.083

CRABP2

8.450

0.819

0.191

0.020

PTGS2

3.794

1.572

0.007

0.585

TGFA

6.383

1.094

0.239

0.001

THBD

0.016

5.127

0.063

0.001

EREG

0.173

4.264

0.101

0.001

AREG

31.412

0.003

0.001

0.001

GPR116

1.429

4.639

0.001

0.021

HLA-DMB

Quantitative PCR results are the mean values from triplicate experiments.

Values were normalized to internal control (L32).
