# Supplementary figures and images for: Transcriptional Profiling of Non-Small Cell Lung Cancer Cells with Activating EGFR Somatic Mutations
Source: PLoS One. 2007 Nov 21;2(11):e1226. doi: 10.1371/journal.pone.0001226 (PMC2080626; doi:10.1371/journal.pone.0001226)

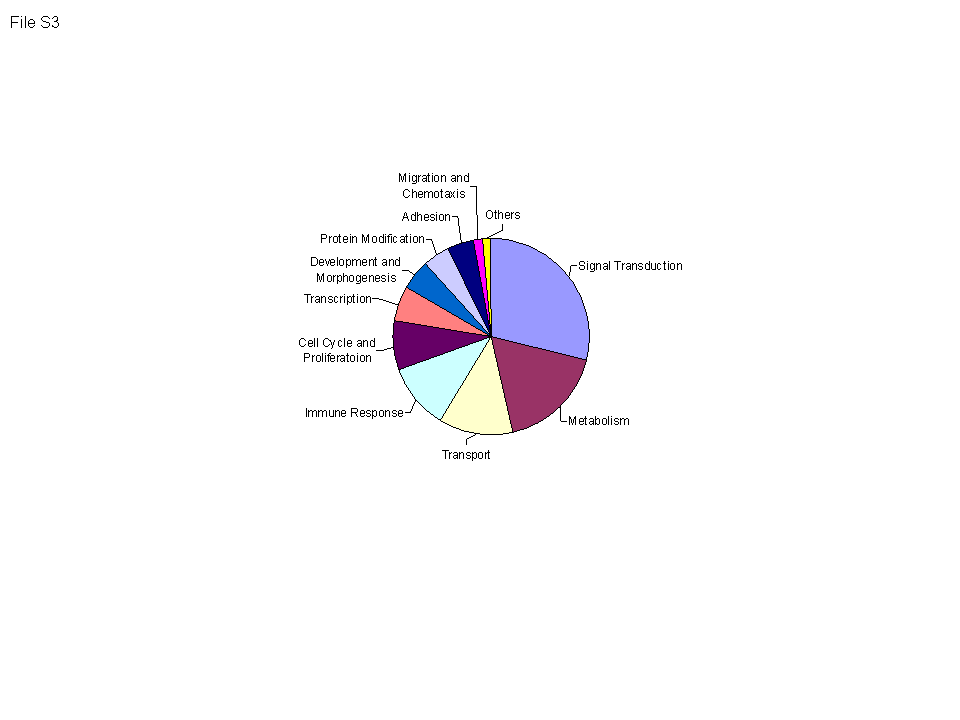

Supplement: File S3 — Mutant EGFR expression profile includes genes with diverse functions. Differentially expressed genes were grouped based on their Gene Ontology functions and represented in a pie chart to illustrate their relative abundance. (0.07 MB TIF) [file pone.0001226.s003.tif]
