# Supplementary material for: Transcriptional Profiling of Non-Small Cell Lung Cancer Cells with Activating EGFR Somatic Mutations
Source: PLoS One. 2007 Nov 21;2(11):e1226. doi: 10.1371/journal.pone.0001226 (PMC2080626; doi:10.1371/journal.pone.0001226)
Supplement: File S6 — Quantitative PCR analysis of selected genes that were differentially expressed in EGFR L858R and δ746-750 NSCLC Cell Lines. Results normalized based on L32 ribosomal RNA expression. (0.08 MB DOC) [file pone.0001226.s006.doc]

File S6. Quantitative PCR Analysis of Selected Genes that were Differentially Expressed in *EGFR* L858R and Δ746-750 NSCLC Cell Lines

**Δ746-750**

**L858R**

**HCC827**

**H4006**

**H3255**

**H1975**

**P value**

**HCC2279**

0.104

0.010

0.036

0.033

0.597

2.636

BMP5

2.957

0.013

3.247

1.365

0.098

0.006

Gal

1.831

0.015

4.306

7.223

0.189

0.524

CARD11

3.720

0.032

1.550

12.814

0.556

0.271

VEGFC

P-value was calculated by performing a one-sided T-test of log-transformed data.
