# Supplementary material for: Transcriptional Profiling of Non-Small Cell Lung Cancer Cells with Activating EGFR Somatic Mutations
Source: PLoS One. 2007 Nov 21;2(11):e1226. doi: 10.1371/journal.pone.0001226 (PMC2080626; doi:10.1371/journal.pone.0001226)
Supplement: File S9 — Quantitative PCR analysis of selected genes that were regulated by gefitinib treatment in TKI-sensitive NSCLC cells (0.09 MB DOC) [file pone.0001226.s009.doc]

File S9. Quantitative PCR Analysis of Selected Genes that were Regulated by Gefitinib Treatment in TKI-sensitive NSCLC Cells

0.83

1.94

2.34

3.04

12.92

4.25

22.49

24.79

1.10

LIPI

1.06

1.24

1.18

2.81

1.81

0.64

2.40

1.082

0.456

CCNG2

0.99

0.52

0.53

1.60

1.39

0.88

1.25

2.05

1.64

ID2

1.03

0.022

0.021

0.60

0.03

0.05

0.34

2.058

6.061

CCL20

0.81

0.765

0.946

0.06

0.309

5.431

0.15

0.145

0.974

DUSP4

1.05

1.210

1.150

0.13

0.143

1.081

0.39

0.327

0.831

LIF

1.00

0.827

0.823

0.02

0.096

4.362

0.14

0.161

1.150

EphA2

0.86

0.392

0.458

2.60

3.494

1.344

2.32

1.988

0.858

EFNA1

1.25

0.135

0.108

0.29

1.631

5.622

0.36

0.619

1.728

FoxD1

0.79

0.116

0.147

<0.01

0.035

8.124

0.03

0.025

0.837

DUSP6

Fold Change

G

D

Fold Change

G

D

Fold Change

G

D

Abbreviations: D, DMSO-treated; G, gefitinib-treated; fold, fold-difference. Quantitative PCR results are the mean values from triplicate experiments. Values were normalized to internal control (L32)

**H1975**

**H4006**

**HCC827**
